# Supplementary material for: Secreted Cytokines within the Urine of AKI Patients Modulate TP53 and SIRT1 Levels in a Human Podocyte Cell Model
Source: Int J Mol Sci. 2023 May 4;24(9):8228. doi: 10.3390/ijms24098228 (PMC10179415; doi:10.3390/ijms24098228)
Supplement: Supplementary file 1 [file ijms-24-08228-s001.zip › ijms-2325266-supplementary.pdf]

Supplemental Material Table of Contents

Table S1: PCR Primers

Table S2: Antibodies

Supplementary Figure S1: Cytokine Array pictures 24 post-surgery.

Supplementary Figure S2: Cytokine Array pictures 72 post-surgery.

Supplementary Figure S3: Quantification of the western blots and full-sized blot images.

Table S1: PCR Primers

Primer name	Sequence	Annealing temperature (°C)	Product length (bp)
RPL0 s	5'-TCGACAATGGCAGCATCTAC-3'	60	195
RPL0 as	5'-ATCCGTCTCCACAGACAAGG-3'		
SIRT1 (Exon 1-2) s	5'-AGGGCGAGGAGGAGGAAGAG-3'	60	122
SIRT1 (Exon 1-2) as	5'-GGCTCTATCCTCCTCATCACTTTC-3'		
TP53 s	5'-CAGGGCAGCTACGGTTTCC-3'	60	157
TP53 as	5'-CAGTTGGCAAAACATCTTGTTGAG-3'		

Table S2: Antibodies

Antigen	Company	Dilution (IF/WB)
Rabbit GAPDH	Cell Signaling #2118	n.a./1:1000
Rabbit pH2A.X (Ser139)	Cell Signaling #9718S	1:200/1:1000
Mouse TP53	Merck Millipore #OP43	n.a./1:1000
Mouse SIRT1	Abcam #ab110304	1:200/1:1000
Anti-mouse Alexa Fluor 488	Invitrogen #A11070	1:500
Anti-mouse HRP-labeled	Thermo Fisher Scientific #NA931	1:4000
Anti-rabbit HRP-labeled	Cell Signaling #7074S	1:1000

AKI stage 2/3

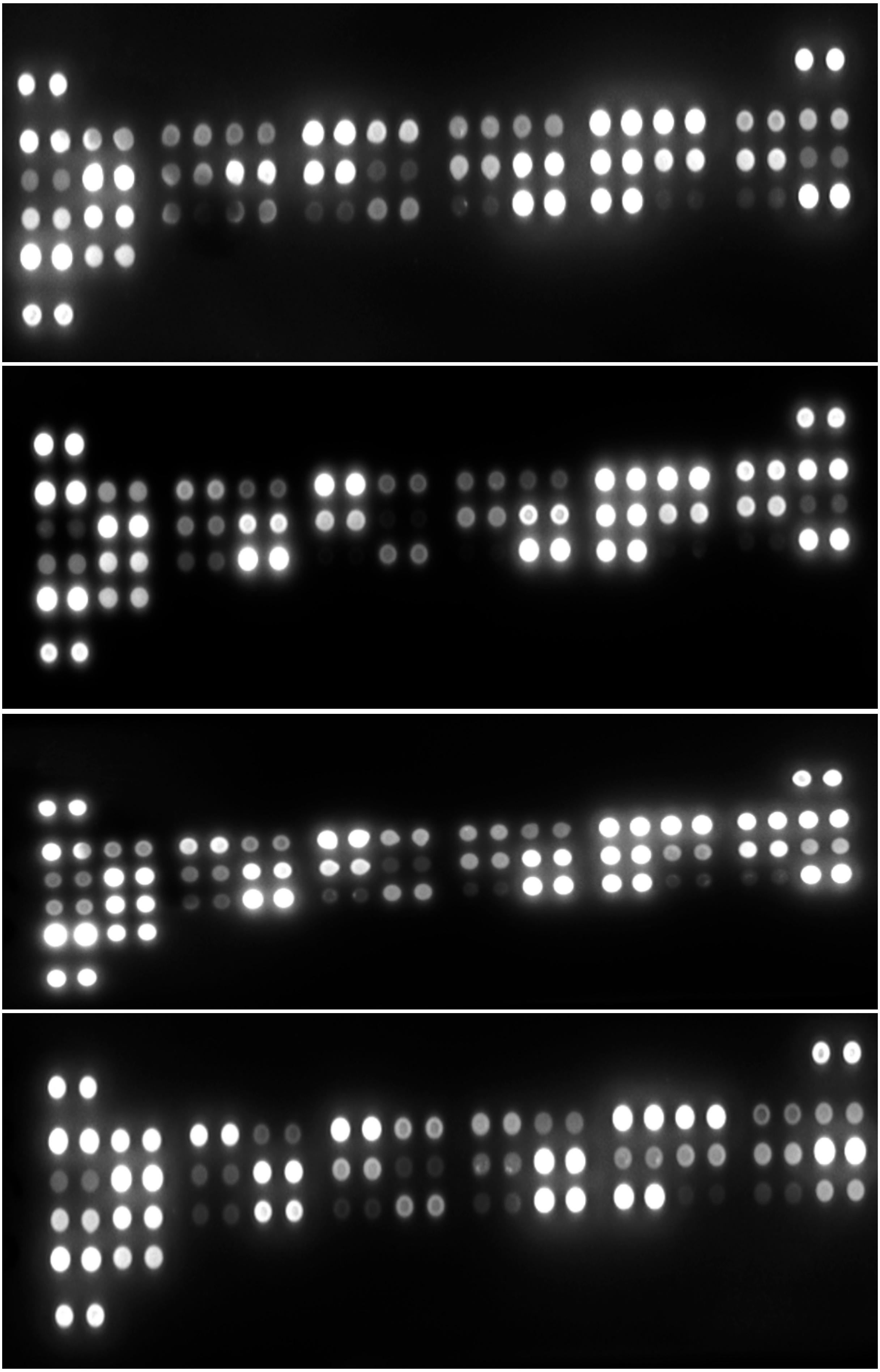

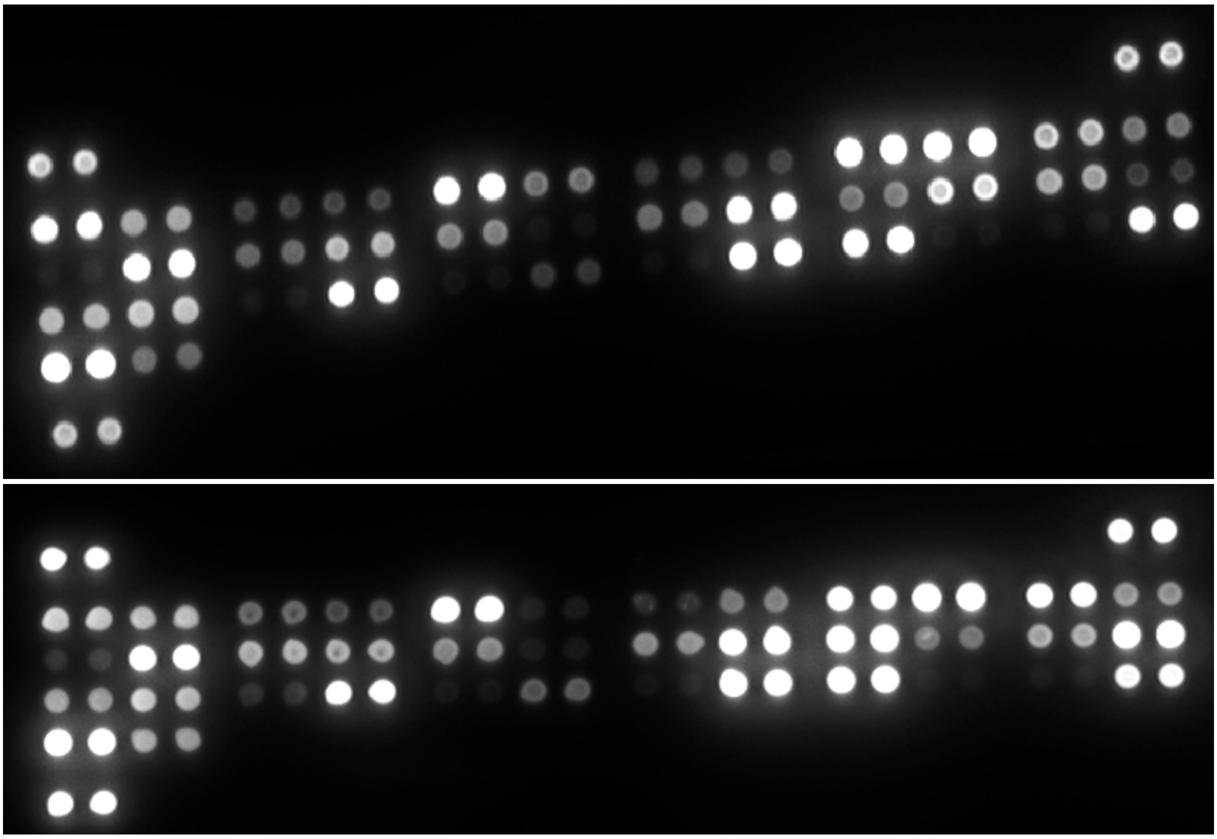

healthy

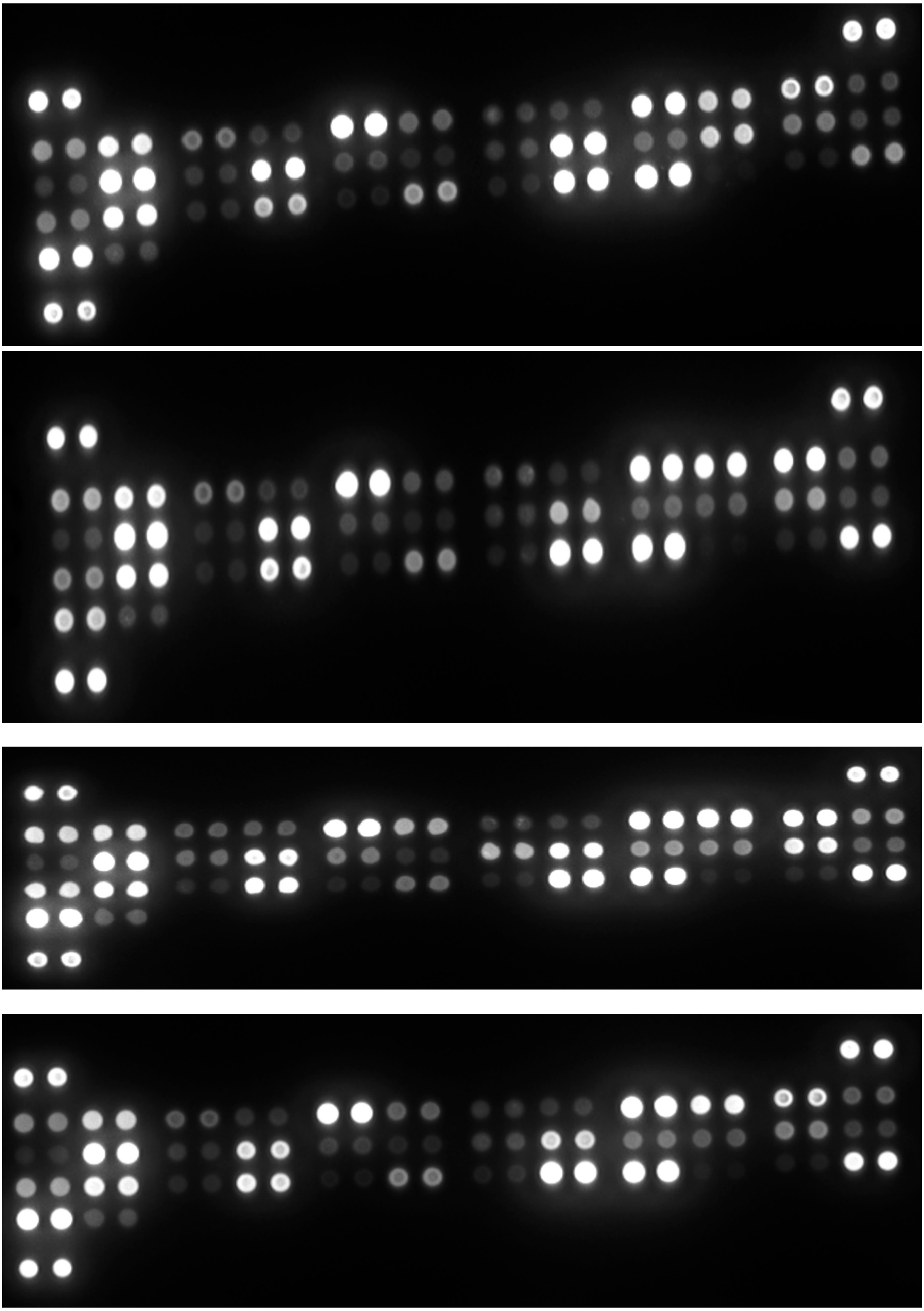

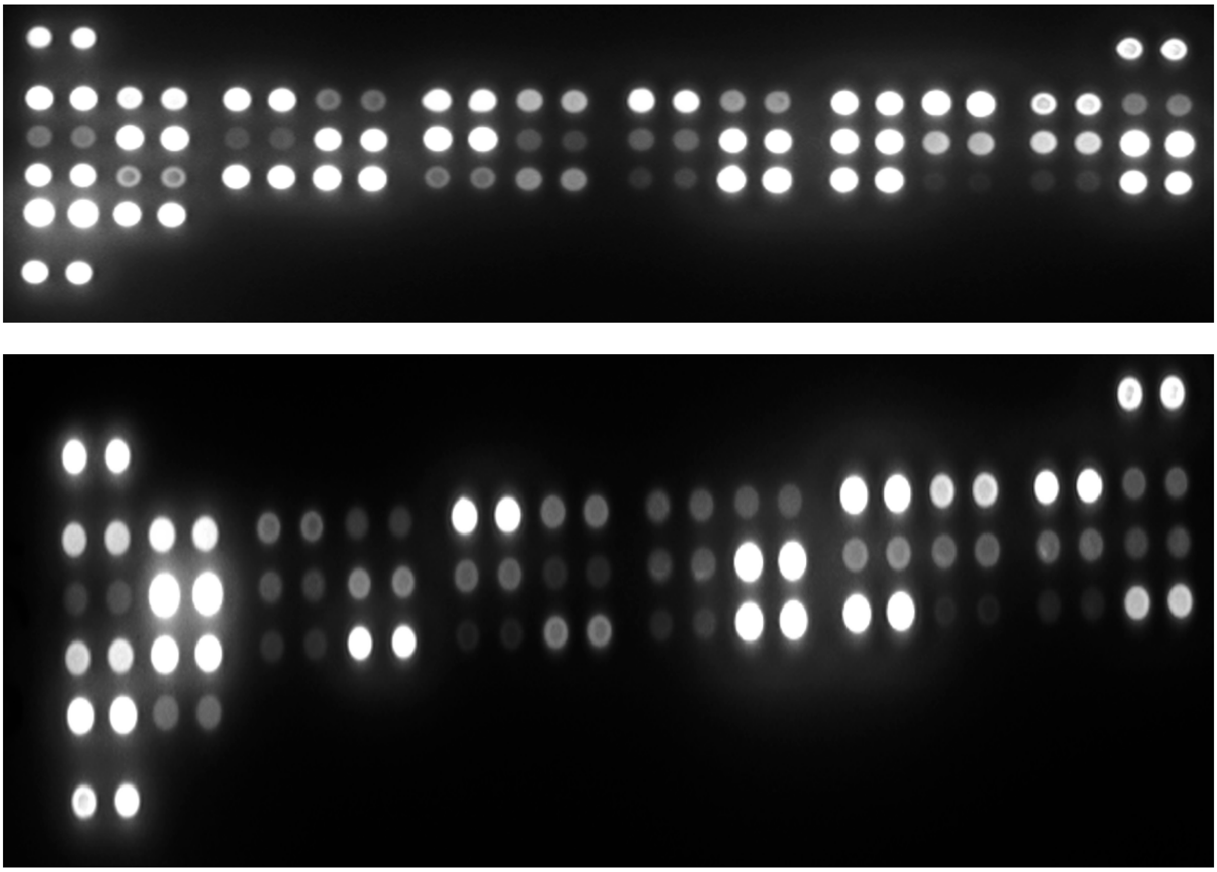

Supplementary Figure S1: Cytokine Array pictures 24 post-surgery.

Pooled healthy

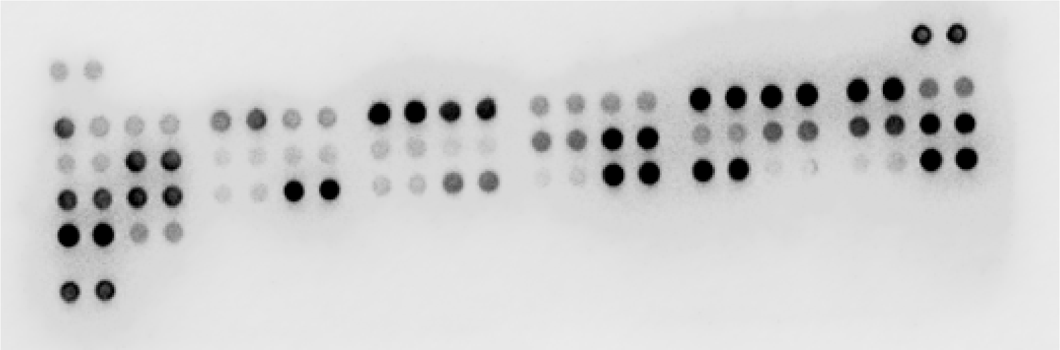

pooled female

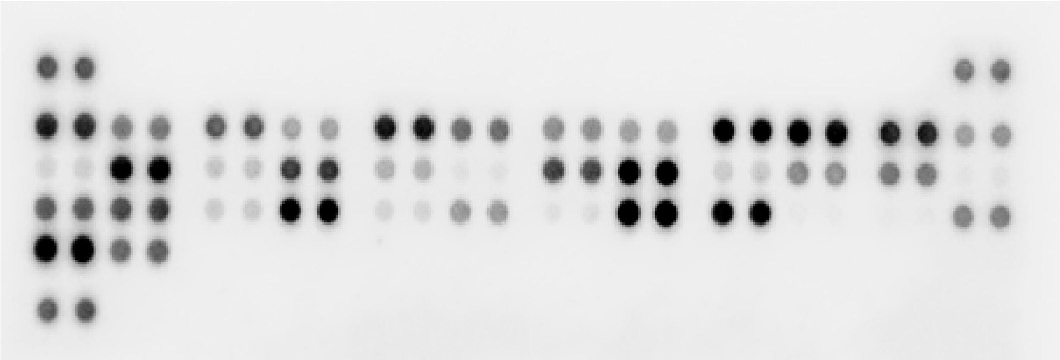

pooled male

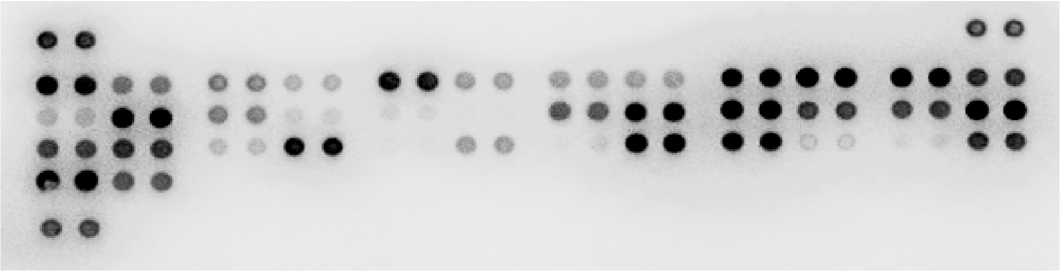

Supplementary Figure S2: Cytokine Array pictures 72 post-surgery.

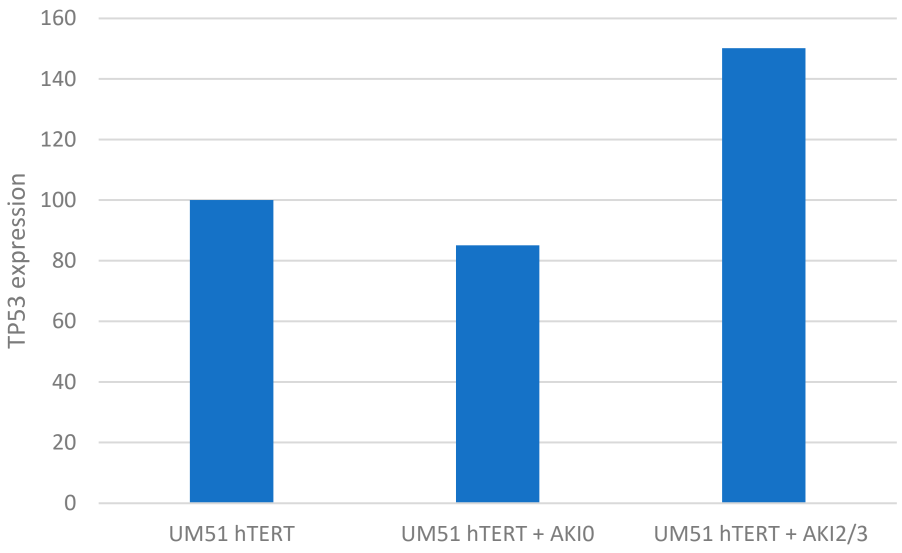
TP53 expression
160
140
120
100
80
60
40
20
0
UM51 hTERT
UM51 hTERT + AKI0
UM51 hTERT + AKI2/3

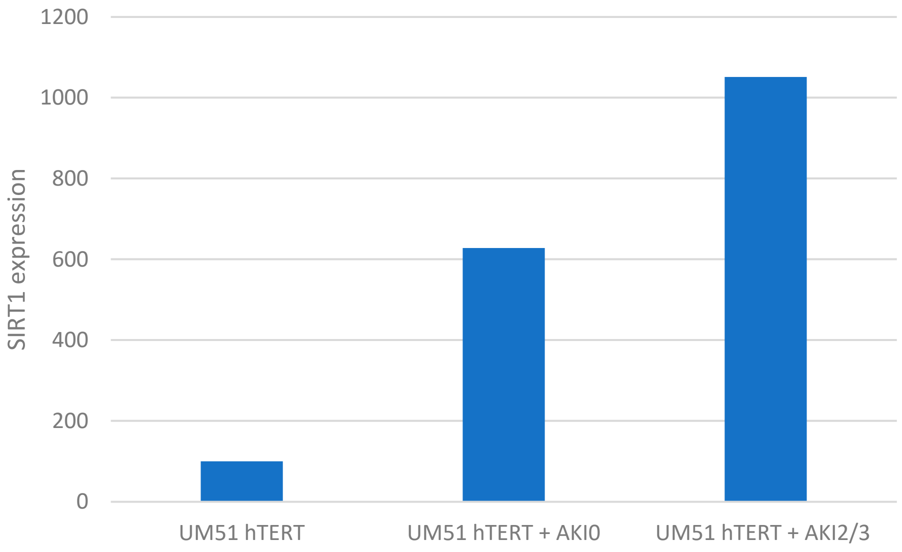
SIRT1 expression
1200
1000
800
600
400
200
0
UM51 hTERT
UM51 hTERT + AKI0
UM51 hTERT + AKI2/3

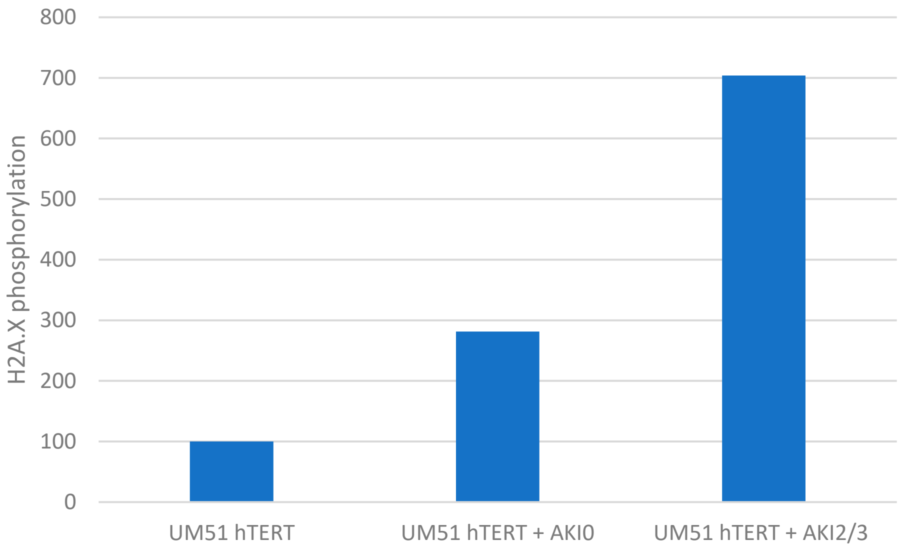
H2A.X phosphorylation
800
700
600
500
400
300
200
100
0
UM51 hTERT
UM51 hTERT + AKI0
UM51 hTERT + AKI2/3

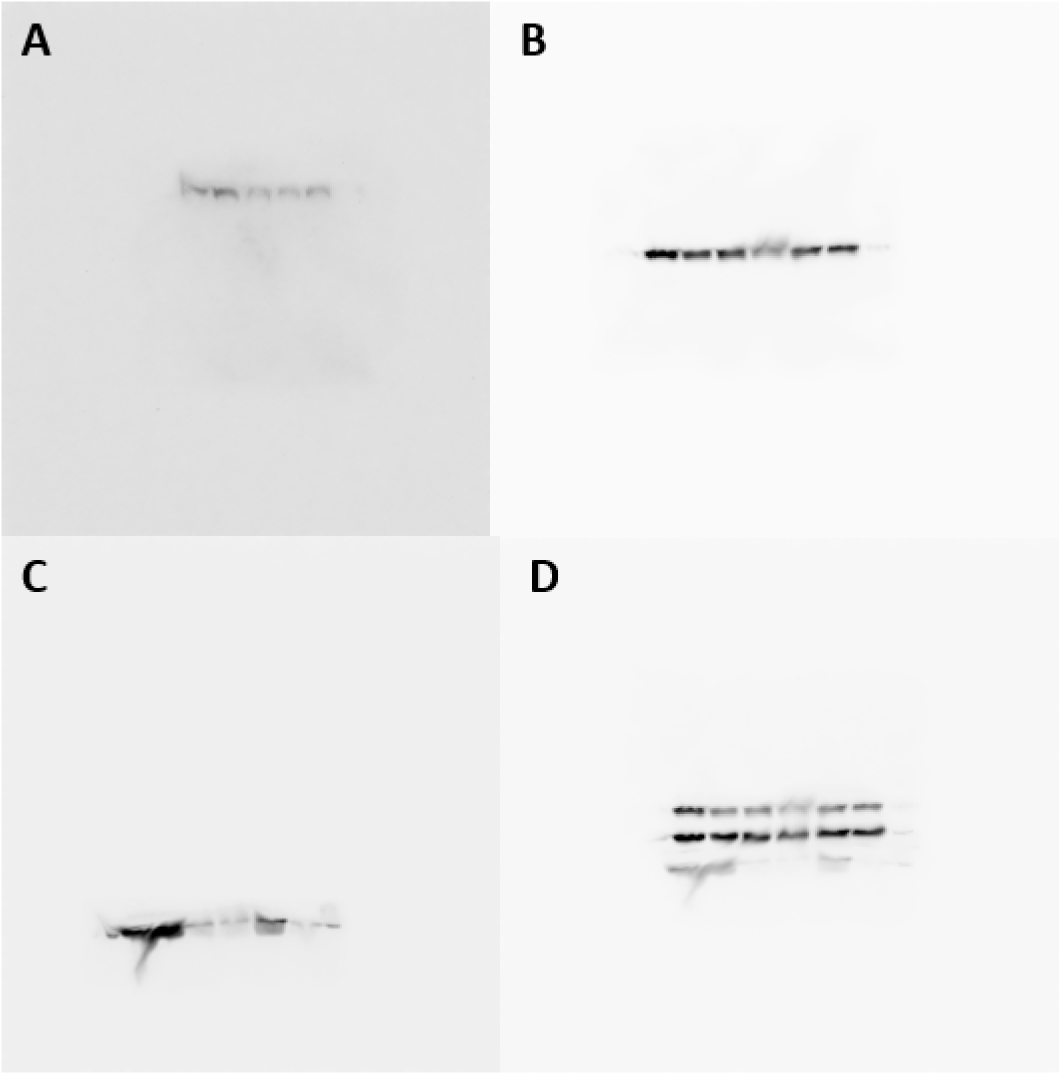

Supplementary Figure S3: Quantification of the western blots and full-sized blot images. Western blot full-sized images are given for SIRT1 (A), TP53 (B), H2A.X (C) and GAPDH (D). UM51-hTERT control podocytes, cultured with urine from AKI0 patients and with urine from AKI 2/3 patients are represented in lane 1, 2 and 5, respectively.
